# Enhancing Postharvest Quality of Fresh-Cut Changgen Mushrooms by Exogenous *L*-Cysteine Treatment: Aspects of Accumulating Amino Acids, Triggering Energy Metabolism and Enhancing Endogenous H_2_S Regulation

**DOI:** 10.3390/foods14030496

**Published:** 2025-02-04

**Authors:** Xingchi Ma, Tianhao Li, Weijian Mao, Yu Zhang, Haoran Liu, Wenwen Jiang, Yanan Sun, Hao Yu, Fansheng Cheng, Dan Zhu

**Affiliations:** 1 College of Food Science and Engineering, Qingdao Agricultural University, Qingdao 266109, China; maxingchi0601@163.com (X.M.); 20212105024@stu.qau.edu (T.L.); maowjian@163.com (W.M.); z17862173918@outlook.com (Y.Z.); haoranliu@stu.qau.edu.can (H.L.); 18351573738@163.com (Y.S.); 2 Qingdao Special Food Research Institute, Qingdao 266109, China; 3 College of Life Science, Qingdao Agricultural University, Qingdao 266109, China; yuhao@qau.edu.cn; 4 Qingdao Institute of Technology, Qingdao 266300, China; 17866845975@163.com

**Keywords:** fresh-cut mushroom, signaling molecule, green preservative, energy status, hydrogen sulfide, preservation, postharvest quality

## Abstract

As a rare and functional edible mushroom, the market potential of ready-to-eat fresh-cut Changgen mushrooms (*Oudemansiella raphanipes*) is booming in developing countries. However, fresh-cut mushrooms preservation is challenging in distribution and consumption. The present study discovered that exogenous *L*-cysteine (*L*-Cys) treatment delayed the weight loss, browning degree, nutrition depletion and microbial contamination of fresh-cut Changgen mushrooms at 4 °C. Based on transcriptomic data, exogenous *L*-Cys significantly activated the metabolism of 17 amino acids, including *L*-Cys and methionine, a prerequisite for hydrogen sulfide (H_2_S) synthesis. Exogenous *L*-Cys also stimulated the activities and gene expressions of cystathionine beta-synthase and cystathionine gamma-lyase, thereby increasing H_2_S levels. Furthermore, exogenous *L*-Cys enhanced the energy metabolism by improving cytochrome c oxidase, H^+^-ATPase and Ca^2+^-ATPase enzymes activity. Exogenous *L*-Cys treatment reduced the reactive oxygen species by regulating enzyme activities such as polyphenol oxidase, catalase and superoxide dismutase. This study contributes valuable insights into the physiological function of *L*-Cys and the role of H_2_S on the fresh-cut Changgen mushroom.

## 1. Introduction

Changgen mushroom (*Oudemansiella raphanipes*), a rare and highly valued edible mushroom, has gained popularity due to the unique flavor and rich nutritional content, including proteins, amino acids, and vitamins [1]. In China, it was originally produced in Yunnan province and was commercially known as Changgen mushrooms. Not until 2016 was its identity revealed, when the species was definitively classified as *O. raphanipes*, thanks to a rigorous examination of both its physical traits and genetic information [2]. The mycelium of Changgen mushrooms is stable on oak sawdust mixed with 5–20% rice bran, with optimal fruiting body production occurring at 5–30% rice bran content [3]. Furthermore, as a ready-to-eat option, fresh-cut Changgen mushrooms have demonstrated potential antioxidant, antitumor, immunomodulatory and hepatoprotective activities [4]. Due to their exceptional health-promoting properties, extensive research has been conducted on the domestication and cultivation conditions, liquid fermentation processes, extraction of bioactive compounds and identification of medicinal functions in Changgen mushrooms [5]. Consequently, the consumption and distribution of these fresh-cut mushrooms have seen a substantial increase in recent years. In 2022, China’s production of Changgen mushrooms reached 40,000 tons, marking an annual growth of 24.28%, significantly contributing to the economic development of rural areas [1]. The de novo genome sequencing data and genetic characteristics of Changgen mushrooms was first published in 2023, which is expected to provide new insights into its physiological study [6].

*L*-cysteine (*L*-Cys), a natural sulfur-containing amino acid, is one of the reagents generally recognized as safe (GRAS) in the food, medicine and cosmetics industries. *L*-Cys is implicated in the biosynthesis of vital biomolecules including antioxidants, vitamins and cofactors [7]. Notably, a previous report discovered that *L*-Cys participated in the resistance of plants to various abiotic stresses and pathogens infections. For example, *L*-Cys treatment effectively prevents postharvest senescence when combined with various organic acids and edible coatings [8]. Additionally, a sodium alginate coating integrated with *L*-Cys and citric acid extended the freshness duration of fresh-cut lotus root for two weeks under refrigerated conditions [9]. Sodium alginate-based edible composite coating materials containing *L*-Cys significantly inhibited the quality deterioration and microbial growth in *Pholiota nameko* mushroom [10]. Meanwhile, an *L*-Cys dose noticeably delays the browning degree of fresh-cut button mushroom *Agaricus bisporus*, and reduces weight loss, the O_2_^−^ generation rate and H_2_O_2_ accumulation [7].

Maintaining a high amino acid content is crucial for maintaining the high nutritional value of mushrooms [11]. The total protein content of Changgen mushrooms can reach up to 32% (*w*/*w*) on a dry weight basis, encompassing 18 amino acids, including all the essential amino acids. In addition to protein biosynthesis, amino acid metabolism plays a vital role in the central metabolism of plants, being involved in secondary metabolite biosynthesis, signal transduction, flavor formation, biotic and abiotic stress [12]. Previously, studies in apple discovered that *L*-Cys treatment increased the cysteine, glutamic acid, serine and leucine level in pulp and peel [13]. The application of 100 mg L^−1^ *L*-Cys prevented plum postharvest brown rot by elevating specific amino acids levels, antioxidant content and the antioxidant enzymes activities of the ascorbate–glutathione pathway [14]. However, the regulation of *L*-Cys on amino acid metabolism in mushroom still needs investigation.

Additionally, *L*-Cys is also a precursor of hydrogen sulfide (H_2_S) biosynthesis, a crucial signaling molecule in many biological functions and disorders in plants and animals. H_2_S is proven to be beneficial in alleviating postharvest senescence and decay in postharvest horticultural plants by modulating the antioxidant defenses [15]. For instance, exogenous H_2_S treatment stimulated the production of endogenous H_2_S and extended the shelf life of banana through a reduction in oxidative stress and the inhibition of the ethylene signaling pathway [16]. Meanwhile, exogenous *L*-Cys treatment can trigger the production of endogenous H_2_S by regulating the enzymatic activities of cystathionine β-synthase (CBS) and cystathionine γ-lyase (CSE) [17]. Furthermore, H_2_S biosynthesis inhibitor propargylglycine treatment on fresh-cut button mushrooms dramatically reverses the beneficial impact of *L*-Cys on the button mushroom [7].

Notably, the core factor contributing to the quality deterioration of horticultural produce lies in the imbalance and deficit of the energy state [18]. Higher ATP and EC levels are beneficial for the inhibition of membrane lipid oxidation and protecting the mitochondrial structure, as well as maintaining good postharvest quality and delaying senescence [19]. The cytochrome c oxidase (CCO), Ca^2+^-ATPase and H^+^-ATPase are integral to energy metabolism in organisms [20]. Studies have reported that H_2_S can mitigate the senescence of postharvest daylilies by maintaining the energy levels in daylily flowers, nectarines and broccoli [21,22,23]. However, the literature remains sparse regarding the regulatory effects of exogenous *L*-Cys on energy metabolism in harvested mushrooms.

These findings suggestedthe potential application of *L*-Cys in the preservation of fresh-cut Changgen mushrooms, although the specific mechanisms remain unclear based on the available data. The present study investigates the effects of *L*-Cys treatment on H_2_S biosynthesis, energy metabolism, amino acid metabolism and reactive oxygen metabolism (ROS) in fresh-cut Changgen mushrooms. Transcriptome analysis will reveal insights into the physiological role of *L*-Cys as a green preservation agent for fresh-cut edible mushrooms.

## 2. Materials and Methods

### 2.1. Materials and Treatments

The Changgen mushrooms were supplied by Shandong Yuanyang Agricultural Development Co. Ltd., Jining, China, and transported to the laboratory immediately after harvest in a temperature-controlled cooler. The mushrooms without mechanical damage with a uniform size and color were selected and their roots were removed to create a tip of about 1.5cm. Mushrooms were randomly divided into 16 treatment groups (90 mushrooms each), each containing 3 repeat groups (30 mushrooms per repeat). The 15 groups of fresh-cut Changgen mushrooms were immersed in 0.125 g L^−1^, 0.25 g L^−1^, 0.5 g L^−1^, 1.0 g L^−1^ and 2.0 g L^−1^ *L*-Cys solutions for 5, 10 and 10 min, while the other group of mushrooms treated with distilled water were used as the control. The treated mushrooms were air-dried in baskets at room temperature for 1 h, packed in PE and stored for 12 days at 4 °C and 90–95% relative humidity. The mushrooms were nondestructively sampled at 0 d, 3 d, 6 d, 9 d and 12 d. The fresh-cut Changgen mushrooms stalk, the main edible tissue, was sampled, frozen in liquid nitrogen and stored under −80 °C for subsequent physiological and biochemical determination.

### 2.2. Determination of Weight Loss, Browning Index (BI) and Colonies Number

The weight loss was determined by the ratio of the sample change to the initial weight [24]. To evaluate the BI, the whiteness (L*), yellowness (b*) and redness (a*) values were determined by randomly measuring the Changgen mushrooms stalk cross-section. The BI was determined according to the previously described method [10]. The colonies number was ascertained employing 3M™ Petrifilm™ aerobic count plates (3M, Maplewood, MN, USA) [25].

### 2.3. Determination of Reducing Sugar, Total Phenols Content and Soluble Protein

The reducing sugar was quantified utilizing the 3,5-dinitrosalicylic acid method, with glucose solution serving as the calibration standard [10]. The methods for determining the total phenolic and soluble protein were referenced from the previously described methods [10]. Total phenolic was measured by the Folin–Ciocalteau colorimetric method and the standard curve was derived from gallic acid. Meanwhile, the soluble protein content was obtained via the Coomassie brilliant blue method, with bovine serum protein serving as the calibration standard. The determined concentrations were expressed as g kg^−1^ based on the fresh sample weight.

### 2.4. Determination of Malondialdehyde (MDA), H_2_O_2_, O_2_^−^ Production Rate and 1,1-Diphenyl-2-Picrylhydrazyl (DPPH) Elimination Ability

The MDA content and H_2_O_2_ content were obtained referring to previously described methods [7]. MDA can undergo a colorimetric reaction with thiobarbituric acid upon heating in acidic conditions, characterized by an absorption peak at 532 nm. The H_2_O_2_ content was quantified using titanium sulfate colorimetry and a standard curve derived from accurate H_2_O_2_ concentrations. The MDA content and H_2_O_2_ content were expressed as mol kg^−1^ of fresh weight. The O_2_^−^ production rate was quantified using the previous method, and expressed as mol kg^−1^ s^−1^ of fresh weight [7].

The DPPH elimination rate was determined according to the methodology described in the preceding study [26]. The Changgen mushrooms samples were homogenized and dissolved in a methanol solution to obtain the extract. Then, two parts of 3 mL the extract and 0.2 mM DPPH were mixed, and the absorbance was observed at 517 nm (A_1_). Next, one volume of deionized water was added to one volume of 0.2 mM DPPH and the extract, respectively, and the absorbance was observed at 517 nm (A_2_, A_3_).

(1) $$\mathrm{DPPH}\;\mathrm{elimination}\;\mathrm{rate}=\frac{A_{2}+A_{3}-A_{1}}{A_{2}}\;\times \;100\%$$

### 2.5. Determination of Antioxidant Enzyme Activities

The polyphenol oxidase (PPO), catalase (CAT) and superoxide dismutase (SOD) activities were determined referring to the previously delineated method [7]. The mushroom extraction buffer was mixed with the guaiacol solution and the H_2_O_2_ solution to determine the peroxidase (POD) activity [27].

The phenylalanine ammonia lyase (PAL) activity was measured using *L*-phenylalanine (20 mM) as a substrate in 50 mM borate buffer at pH 9 [28]. The glutathione reductase (GR), ascorbate peroxidase (APX) and lipoxygenase (LOX) activity were assessed using previously reported methods [29,30]. The enzyme activities were expressed as U kg^−1^ of protein.

### 2.6. Determination of Endogenous H_2_S, CBS and CSE Enzyme Activities

The endogenous H_2_S was quantified employing a modified version of the established method [7]. A total of 2.0 g of Changgen mushrooms were homogenized in potassium phosphate buffer (100 mM PBS, 10 mM EDTA, pH 6.8). Next, the supernatant was transferred to buffer (10 mM EDTA, 100 mM PBS and 0.2 mM 2-nitrobenzoic acid, pH 6.8). The absorbance at 412 nm was measured following centrifugation and incubation. The H_2_S concentration was expressed in terms of mol kg^−1^ fresh weight, with the accurate NaHS solution severing as the standard substance.

The CBS and CSE activities were measured by the previously established method [17]. The method for assessing CSE activity mirrored that of CBS, with the exception of substituting *L*-Cys with an equivalent concentration of homocysteine. The enzyme activities were expressed as U kg^−1^ of fresh weight.

### 2.7. Determination of CCO, Ca^2+^-ATPase and H^+^-ATPase Enzymes

The mitochondrial extraction occurred using a previously described method [31]. The Changgen mushroom samples were homogenized in a pre-cooled 100 mM Tris-HCl buffer (1.0 mM ethylene diamine tetraacetic acid, 250 mM sucrose and 300 mM mannitol, pH 7.5). The precipitate was rinsed and suspended in the abovementioned 10 mM Tris-HCl buffer after centrifuging. The resulting suspension constituted crude mitochondrial extract by differential centrifugation. The activity of three enzymes was measured using a previously delineated method [32]. The CCO activity was expressed as U kg^−1^ protein, where one unit denoted a 0.01 absorbance change at 600 nm per second. The ATPase activity was shown as U kg^−1^ protein, where one unit represented the discharge of 1.0 mol of phosphorus at 660 nm per hour.

### 2.8. Determination of Adenosine Triphosphate (ATP), Adenosine Diphosphate (ADP), Adenosine Monophosphate (AMP) and Energy Charge (EC)

The ATP, ADP and AMP levels were quantified utilizing a method outlined in prior research by an HPLC equipped with an XBridge C_18_ reverse-phase column [33]. AMP, ADP and ATP levels were expressed as g kg^−1^ of fresh weight, which was calculated by an external standard curve.

(2) $$\mathrm{EC}\;\mathrm{value}=\frac{ATP+1/2\times ADP}{ATP+ADP+AMP}$$

### 2.9. Determination of Free Amino Acids

Amino acids levels were measured by a previously reported procedure [14]. The ninhydrin derivatization was performed to assess the amino acids levels using an amino acid analyzer (Hitachi LA8080, Tokyo, Japan). The identification of amino acid types was accomplished through the peak plot of the standard solutions, and the quantification was carried out based on the peak area. The levels were expressed as g kg^−1^ fresh weight.

### 2.10. Transcriptome Sequencing and Analysis

Three biological samples from both treatment groups at 3 d were transferred to Biomarker Technologies (Beijing, China) for transcriptome sequencing. The qualified mRNA was enriched and interrupted. Then, the first-strand and second-strand cDNA were assembled and attached to sequencing splices for size selection. The PCR-amplified cDNA library was sequenced via the PE150 mode of an Illumina NovaSeq 6000 platform. After sequence alignment with the designated reference genome (https://ngdc.cncb.ac.cn/gwh) (accessed on 1 June 2022) to obtain mapped data, library quality assessment, differential expression analysis, function annotation and enrichment of metabolic pathways were conducted.

Transcriptome sequencing technology can comprehensively and rapidly obtain the sequence information of mRNA transcribed in a specific tissue of a species in a certain state, thereby uncovering the molecular mechanisms underlying specific biological processes.

The total RNA extraction from Changgen mushrooms tissue was performed using a plant RNAexr kit (Huayueyang Biotechnology, Beijing, China). Moreover, real-time PCR (qRT-PCR) was performed using the QuantStudio 3 (Applied Biosystems, Foster City, CA, USA). The relative target gene expression was assessed by the 2^−∆∆CT^ method. Table 1 lists the primers utilized in this experiment, with *AbEF1-a* chosen as the reference gene.

### 2.11. Statistical Analysis

All experiments were performed in three independent biological replicates. The results are presented as the mean ± standard deviation (SD). To assess significant variations in mean values between the control and *L*-Cys treatment groups, a one-way analysis of variance (ANOVA) was conducted using SPSS 26.0 software (SPSS, Inc., Chicago, IL, USA). The data were compared using Duncan’s new multiple range test at *p* < 0.05, utilizing DPS 2022 (DPS, Data Processing System, Beijing, China). The figures were drawn according to the means ± SD, using Origin 2023 (OriginLab Corporation, Northampton, MA, USA).

## 3. Results

### 3.1. L-Cys Prevented Weight Reduction, Browning and Microbial Contamination

**Figure 1:**
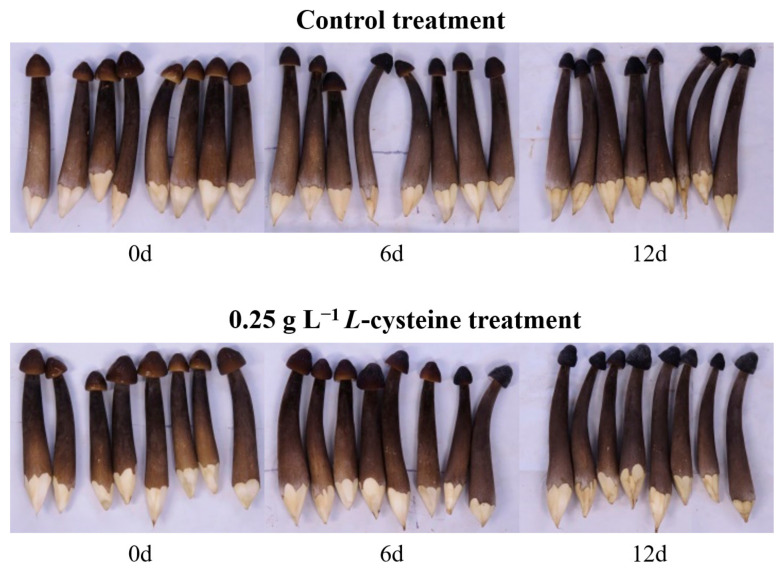
The appearance quality of differentially treated fresh-cut Changgen mushrooms. During the storage period, time points of 0, 6 and 12 d were selected to show the appearance difference between 0.25 g L^−1^ *L*-Cys treatment and the control treatment.

According to the effect of the gradient preliminary experiment, the optimal treatment condition was 0.25 g L^−1^ *L*-Cys solutions for 10 min of fresh-cut Changgen mushrooms. As shown in Figure 1, the fresh-cut Changgen mushrooms, characterized by their black skin and white inner tissue, showed minimal changes in the epidermal layer during storage. However, the cap and the exposed subcutaneous tissue progressively discolored brown. Compared with the control treatment, the browning of fresh-cut Changgen mushrooms treated with *L*-Cys was significantly less with the increase in storage time.

**Figure 2:**
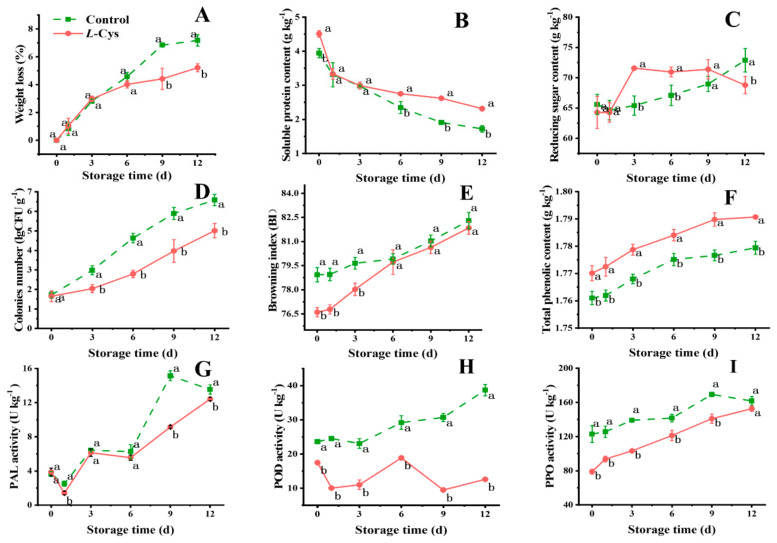
Changes in weight loss (**A**), soluble protein (**B**), reducing sugar (**C**), colonies number (**D**), BI (**E**), total phenolic (**F**), PAL activity (**G**), POD activity (**H**) and PPO activity (**I**) of differentially treated fresh-cut Changgen mushrooms. The horizontal coordinate indicates the storage time, and the vertical coordinate indicates the value of an indicator. The solid red line represents the *L*-Cys treatment, and the dotted green line represents the control treatment. Bars represent standard deviation (±SD). The same letters represent no significance, and different letters represent statistically significant differences (*p* < 0.05).

During storage, the water loss of fresh-cut Changgen mushrooms increased (Figure 2A). The weight loss observed in the *L*-Cys group was markedly less compared to the control group from 9 d (*p* < 0.05), indicating that *L*-Cys treatment retained more moisture during the later storage period. The BI of the *L*-Cys treatment group was consistently lower throughout storage, exhibiting significant differences before 2 d (*p* < 0.05) (Figure 2E). This indicates that *L*-Cys treatment effectively mitigates the browning of fresh-cut Changgen mushrooms. Microbial contamination is a critical parameter for the ready-to-eat food. A high colonies number will increase the risk of foodborne illness. In the present study, the colonies number in the *L*-Cys treatment group exhibited a significantly slower rise compared with the control group (Figure 2D).

### 3.2. L-Cys Preserved Reducing Sugars, Soluble Proteins and Total Phenols Content

The soluble protein content in the two groups decreased with the storage period (Figure 2B). Meanwhile, the soluble protein content of the *L*-Cys treatment group almost maintained a higher level than the other group (*p* < 0.05). Regarding the reducing sugar content, both groups exhibited stability throughout the entire storage period (Figure 2C). Notably, from 3 d to 9 d, the *L*-Cys group displayed a statistically significant higher reducing sugar level than the control group (*p* < 0.05). As depicted in Figure 2F, the total phenol content increased with the storage time in both groups. Importantly, the *L*-Cys group significantly maintained a higher total phenolic level throughout storage duration (*p* < 0.05).

### 3.3. L-Cys Inhibited the Lipid Oxidation

**Figure 3:**
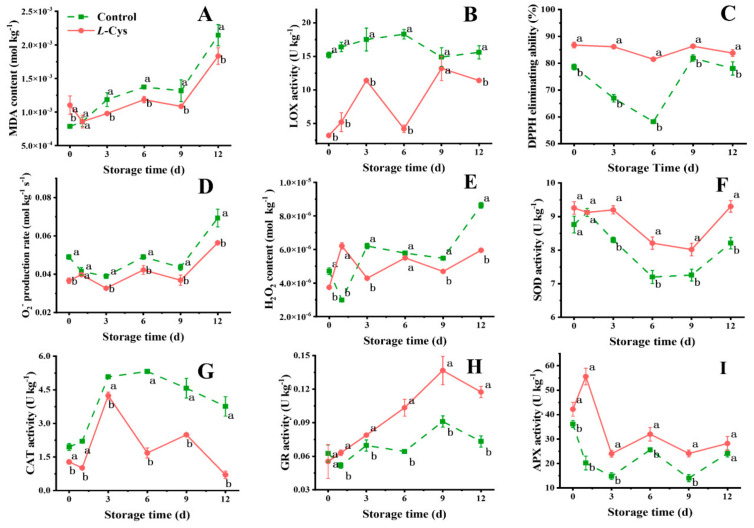
Changes in MDA (**A**), LOX activity (**B**), DPPH eliminating ability (**C**), O_2_^−^ production rate (**D**), H_2_O_2_ content (**E**), SOD activity (**F**), CAT activity (**G**), GR activity (**H**) and APX activity (**I**) of differentially treated fresh-cut Changgen mushrooms. The horizontal coordinate indicates the storage time, and the vertical coordinate indicates the value of an indicator. The solid red line represents the *L*-Cys treatment, and the dotted green line represents the control treatment. Bars represent standard deviation (±SD). The same letters represent no significance, and different letters represent statistically significant differences (*p* < 0.05).

The MDA levels, an indicator of lipid peroxidation, exhibited an overall upward trend with the duration of storage (Figure 3A). Compared with the control group, *L*-Cys treatment significantly mitigated the rise in MDA levels during the middle and late storage stages (*p* < 0.05).

LOX activity, which is responsible for the oxidation of unsaturated fatty acids, exhibited gradual fluctuations in the *L*-Cys group throughout the storage period (Figure 3B). The *L*-Cys group demonstrated a significant decrease in LOX activity, characterized by a bimodal pattern (*p* < 0.05).

### 3.4. L-Cys Regulated the Metabolism of ROS

Although the O_2_^−^ production rate fluctuated in both groups as the storage period extended, the *L*-Cys group showed a significantly lower rate (*p* < 0.05) (Figure 3D). The H_2_O_2_ content increased in two groups with increasing storage time (Figure 3E). However, the *L*-Cys treatment group subsequently declined to a level that was significantly lower than that of control group (*p* < 0.05). The DPPH scavenging ability, indicative of antioxidant capacity, reached a valley value at 6 d for both groups (Figure 3C). Throughout the storage period, the *L*-Cys group demonstrated a significantly higher DPPH scavenging ability compared to the control group (*p* < 0.05).

The CAT activity in the *L*-Cys treated group presented a bimodal pattern during storage, with peak levels observed at 3 d and 9 d, respectively (Figure 3G). In contrast, the control group’s CAT activity peaked at 6 d, followed by a decline. The *L*-Cys group consistently exhibited lower CAT activity throughout the storage period (*p* < 0.05). The SOD activity initially decreased in the two groups, followed by an increase. Meanwhile, the *L*-Cys group remained consistently higher compared to the control group throughout storage (*p* < 0.05) (Figure 3F). As to the POD activity, the control group displayed a gradual increase over time (Figure 2H). Conversely, the *L*-Cys group exhibited consistently lower POD activity compared to the control group throughout storage, with significant reductions of 69.1% at 9 d and 67.4% at 12 d, respectively (*p* < 0.05). Furthermore, *L*-Cys treatment enhanced the activities of GR and APX (Figure 3H,I), while it reduced the activities of PAL and PPO (Figure 2G,I). Therefore, *L*-Cys treatment proved highly effective in regulating the ROS during the storage of fresh-cut Changgen mushrooms.

### 3.5. L-Cys Regulated the Amino Acids Metabolism

**Figure 4:**
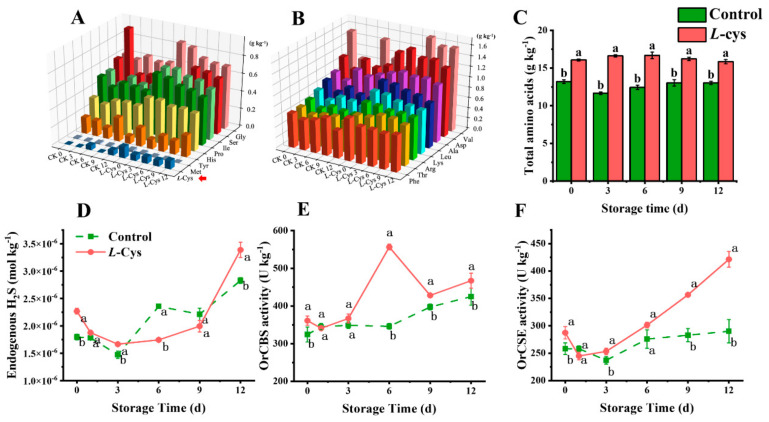
Changes in 16 amino acids (**A**,**B**), total amino acids (**C**), endogenous H_2_S content (**D**), OrCBS activity (**E**) and OrCSE activity (**F**) of differentially treated fresh-cut Changgen mushrooms. The X axis indicates the storage time of different treatments, the Y-axis indicates the class of amino acids and the Z axis indicates the content of various amino acids. The amino acids indicated by the red arrow are sulfur-containing amino acids, which are essential precursors for H_2_S synthesis. (**A**,**B**). The horizontal coordinate indicates the storage time, and the vertical coordinate indicates the value of an indicator. The red column represents the *L*-Cys treatment, and the green column represents the control treatment (**C**). The solid red line represents the *L*-Cys treatment, and the dotted green line represents the control treatment (**D**–**F**). Bars represent standard deviation (±SD). The same letters represent no significance, and different letters represent statistically significant differences (*p* < 0.05).

Throughout the storage period, 17 amino acids in both the control and *L*-Cys treatment groups were detected. Glutamate (Glu) was the most abundant amino acid, and the remaining 16 amino acids are shown in Figure 4A,B. Collectively, the application of *L*-Cys resulted in elevated levels of all measured amino acids. Notably, Glu, valine (Val) and aspartate (Asp) were the three most abundant amino acids in the dry weight of the fresh-cut Changgen mushrooms, peaking at 3 d. Conversely, methionine (Met), *L*-Cys and tyrosine (Tyr) were represented in the last three proportions at the same time point. These findings verified the fact that *L*-Cys affected the total amino acids levels in fresh-cut Changgen mushrooms (Figure 4C).

### 3.6. The Role of L-Cys in Stimulating the Biosynthesis of H_2_S

As depicted in Figure 4A, *L*-Cys significantly increased the content of sulfur-containing amino acids *L*-Cys and Met, which indicated that the *L*-Cys may significantly regulate the downstream H_2_S biosynthesis.

The activity of the cystathionine beta-synthase (OrCBS) showed a gradual increase in the control group over the storage period, whereas the *L*-Cys treatment group displayed a notable peak at 6 d (Figure 4E). From 3 d onwards, the OrCBS activity in the *L*-Cys-treated group significantly surpassed that of the control (*p* < 0.05). Conversely, the cystathionine gamma-lyase (OrCSE) enzyme activity declined initially in the two groups, followed by an increase. Meanwhile, the OrCSE activities in *L*-Cys group consistently exceeded the control group (*p* < 0.05) (Figure 4F).

The endogenous H_2_S level in the *L*-Cys group declined initially, followed by an increase throughout the storage period (Figure 4D). In contrast, H_2_S levels in the control group fluctuated with an increasing trend, only surpassing those of the *L*-Cys group at 6 d. Therefore, *L*-Cys treatment can significantly increase the H_2_S content at the pre-storage stage.

### 3.7. The Role of L-Cys in Regulating the Energy Metabolism

**Figure 5:**
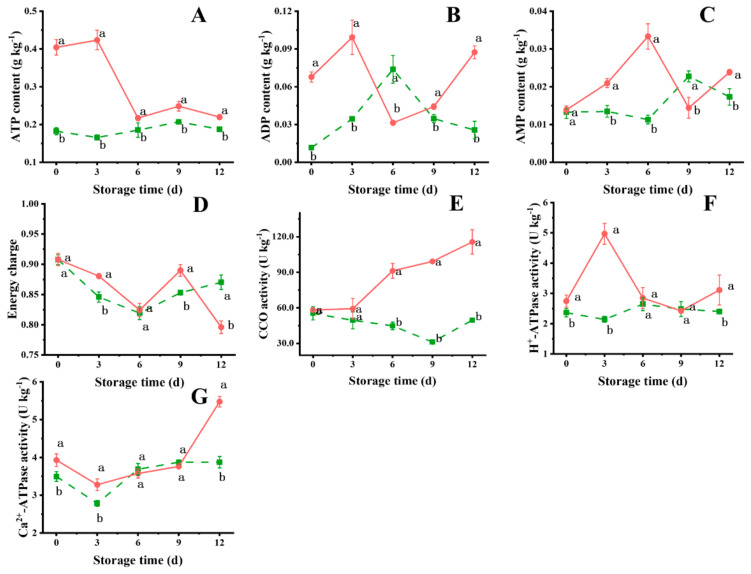
Changes in ATP content (**A**), ADP content (**B**), AMP content (**C**), EC level (**D**), CCO activity (**E**), H^+^-ATPase activity (**F**) and Ca^2+^-ATPase activity (**G**) of differentially treated fresh-cut Changgen mushrooms. The horizontal coordinate indicates the storage time, and the vertical coordinate indicates the value of an indicator. The solid red line represents the *L*-Cys treatment, and the dotted green line represents the control treatment. Bars represent standard deviation (±SD). The same letters represent no significance, and different letters represent statistically significant differences (*p* < 0.05).

As shown in Figure 5A, the ATP content in the *L*-Cys group was at a high level for the initial 3 d, followed by a dramatically decreasing trend, while the control group exhibited minor fluctuations and remained at a significantly low level (*p* < 0.05). Both the control and *L*-Cys groups displayed significant fluctuations in ADP content as their storage time progressed (Figure 5B). A peak value was observed in the control group at 6 d, whereas the *L*-Cys group displayed a trough. The ADP content in the treatment group significantly exceeded that of the control group for the majority of the storage period (*p* < 0.05). The AMP content in the *L*-Cys group increased gradually before 6 d of storage, followed by a decrease at 9 d. Conversely, the control group demonstrated a trough at 6 d and a peak at 9 d. A substantial difference in AMP content was observed between the two groups (*p* < 0.05), with the *L*-Cys group consistently having higher levels, except on 9 d (Figure 5C). Except at 12 d, the EC of the *L*-Cys group remained a high level throughout the storage, suggesting that the *L*-Cys treatment mitigated energy loss in fresh-cut Changgen mushrooms (Figure 5D).

The CCO activity declined in the control group, while the *L*-Cys treatment demonstrated a contrary tendency and CCO activity was significantly enhanced (*p* < 0.05) (Figure 5E). The H^+^-ATPase activity in the control group remained at a stable level during the storage, while the treatment group displayed a considerable fluctuation, reaching a maximum point at 3 d (Figure 5F). The Ca^2+^-ATPase activity in the two groups displayed a similar trend, and the *L*-Cys group was substantially higher than that of control group (*p* < 0.05) (Figure 5G).

### 3.8. The Transcriptome Analysis and qRT-PCR Verification of the L-Cys Treatment

**Figure 6:**
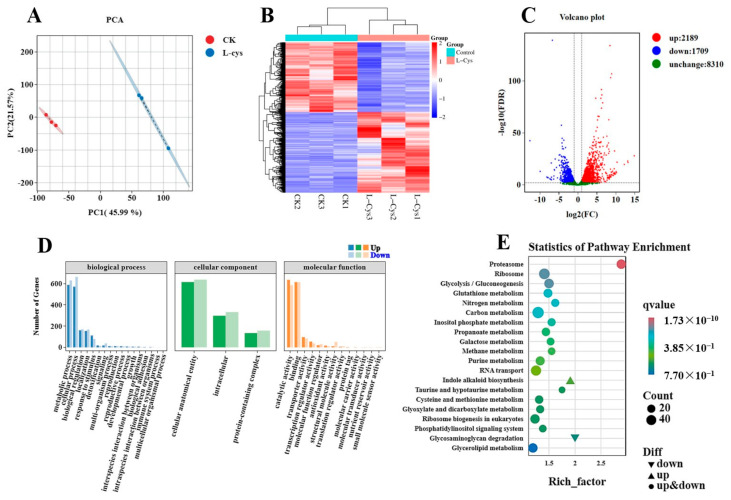
Transcriptomic analysis of the Changgen mushrooms by different treatments. (**A**) PCA of the two principal components (PC1 and PC2). Different coordinates represent different principal components, and percentage represents the contribution values of corresponding principal components to sample differences; (**B**) Heatmap illustrating the overall difference in gene expression in different treatments. The horizontal coordinate represents the sample name and the clustering result of the sample, and the vertical coordinate represents the differential gene and the clustering result of the gene; (**C**) Volcano plots of the DEGs. The horizontal coordinate represents the logarithmic value of the difference multiple of DEGs. The ordinate represents the negative value of the statistical significance of the change in gene expression.; (**D**) GO enrichment analysis. The horizontal axis represents the enrichment of metabolic pathways in the biological process, cellular component and molecular function, and the vertical axis represents the number of genes corresponding to metabolic pathways; (**E**) KEGG enrichment analysis. Abscissa represents rich factor, and ordinate represents the KEGG enrichment pathways.

The transcriptome analysis was conducted to identify differentially expressed genes (DEGs) between the *L*-Cys and control samples. Principal component analysis (PCA) analysis showed a distinct separation between the groups, as depicted in the PC1 and PC2 score plots, signifying a high level of replication correlation (Figure 6A). The heatmap further illustrated a significant disparity in the DEGs profiles between the two groups, with a discernible consistency across biological replicates (Figure 6B). A total of 3898 DEGs were identified, with 2198 up-regulated and 1709 down-regulated genes (Figure 6C). The GO enrichment analysis was performed to examine the biological functions associated with the DEGs. The results indicated that exogenous *L*-Cys regulated the antioxidant activity, nutrient reservoir activity and small molecular sensor activity in Changgen mushrooms (Figure 6D). KEGG pathway enrichment analysis elucidated the most significantly enriched pathways, including proteasome, carbon metabolism, cysteine and methionine metabolism (Figure 6E).

**Figure 7:**
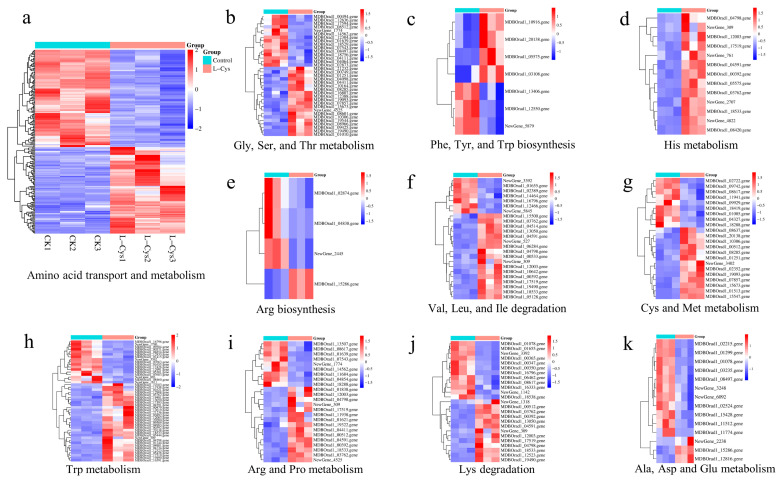
Heatmap of gene expressions of the amino acid metabolism (**a**); Gly, Ser and Thr metabolism (**b**); Phe, Tyr and Trp biosynthesis (**c**); His metabolism (**d**); Arg biosynthesis (**e**); Val, Leu and Ile degradation (**f**); Cys and Met metabolism (**g**); Trp metabolism (**h**); Arg and Pro metabolism (**i**); Lys degradation (**j**); Ala, Asp and Glu metabolism (**k**). The horizontal coordinate represents the sample name and the clustering result of the sample, and the vertical coordinate represents the differential gene and the clustering result of the gene.

A total of 189 DEGs were involved in amino acid transport and metabolism (Figure 7a), which were categorized into 10 distinct groups (Figure 7b–k). There were also 26 DEGs enriched in Cys and Met metabolism (Figure 7g). Meanwhile, the tryptophan and histidine metabolic pathways enriched 53 and 13 DEGs, respectively (Figure 7d and Figure 7h). These transcriptional regulation findings further validate the influence of *L*-Cys on amino acid content.

Notably, two key genes encoding H_2_S biosynthesis were chosen for validation through qRT-PCR, as listed in Table 1. The expression levels of *OrCBS* and *OrCSE* were up-regulated, aligning with the transcriptome data and the enzyme activity data, as previously described.

Additionally, the study identified 22 DEGs involved in energy production and conversion, with 18 associated with oxidative phosphorylation. Among these, five key genes encoding COO, H^+^-ATPase and Ca^2+^-ATPase enzymes were validated using qRT-PCR (Table 1). The genes encoding COO and Ca^2+^-ATPase were up-regulated, whereas H^+^-ATPase genes 1, 2 and 3 were down-regulated. These findings reinforce the previous enzyme activity and energy substance data related to energy metabolism.

Furthermore, the transcriptional regulation of ROS was also notably significant (Table S1). The *LOX1*, *LOX2*, *PAL*, *POD1* and *POD2* genes were down-regulated, whereas the *CAT1-3*, *SOD1*, *SOD2* and *GR* genes were up-regulated. These findings are congruent with previous studies highlighting their roles in antioxidant defense. Additionally, genes involved in cell wall/membrane/envelope biogenesis were down-regulated, including *mannosyl-oligosaccharide 1, 2-alpha-mannosidase1*, *mannosyl-oligosaccharide 1, 2-alpha-mannosidase2*, *endo-1, 3-beta-glucosidase* and *chitinase* genes. These genes are implicated in cell wall metabolism and responses to dehydration (Table S1).

## 4. Discussion

Similarly to many other commercially cultivated mushrooms, harvested Changgen mushrooms experience rapid senescence and nutrition loss. If fresh-cut Changgen mushrooms are not promptly and adequately precooled, this process is exacerbated. This phenomenon is attributed to its high cultivation temperature, vigorous respiratory rate and high water content [34]. In addition, due to the destroyed integrity and the absence of a protective cuticle layer, fresh-cut Changgen mushrooms are subjected to severe nutritional loss, flavor, taste and microbial infection. Consequently, the short freshness duration imposed significant constraints on its consumption, geographical distribution and even risks to human health [35,36]. Thus, there is an urgent need to develop green and efficient preservation technologies for fresh-cut Changgen mushrooms.

Fresh-cut mushrooms are highly perishable and prone to rapid quality deterioration, including discoloration, moisture loss, increase in microbial quantity, loss of nutrition and flavor [37]. These quality changes significantly impact the market value and consumer acceptance of mushrooms. In the present study, the application of *L*-Cys effectively decelerated these negative changes, preserving water content, reducing sugars, soluble proteins and total phenol levels, while also mitigating browning and reducing microbial colony counts (Figure 2A–F). These outcomes fully supported the beneficial impacts of *L*-Cys on fresh-cut Changgen mushrooms, and reduce the associated risks of ready-to-eat vegetables. Similar effects of *L*-Cys in delaying weight loss and reducing microbial colony counts have been observed in other fresh-cut produce, such as button mushrooms and litchi [7,8].

The senescence process in mushrooms is accompanied by the loss of cell membrane functionality. This study found that *L*-Cys decreased the LOX activity, reduced the MDA content and alleviated the soft tissue damage in mushrooms (Figure 3A,B). These findings suggest that the degradation of unsaturated fatty acids is delayed, and *L*-Cys treatment is beneficial for preserving the cell membrane integrity of fresh-cut Changgen mushrooms, which is attributed to the inherent reducing properties of *L*-Cys, as observed in fresh-cut *A. bisporus* [7].

Browning, a critical factor affecting mushroom quality, results from the oxidation and polymerization of phenolic compounds, and is associated with PAL, PPO and POD activity [7]. *L*-Cys treatment significantly retarded the browning process, which is consistent with the preservation of total phenol levels and the inhibition of PAL, PPO and POD activities during storage (Figure 2F–I). The PAL controls the biosynthesis of various secondary phenolic compounds and regulates the browning process of edible mushroom [38]. The PAL enzyme, which controls the biosynthesis of secondary phenolic compounds, showed increased activity with extended storage periods in fresh-cut Changgen mushrooms. However, *L*-Cys treatment significantly lowered PAL activity at later storage stages, retarding lignification and polyphenol metabolism, which was in accordance with the results in broccoli [39]. Additionally, the POD and PPO, participating in the phenolic oxidization, are the key factors contributing to postharvest browning [40]. The present study discovered that the *L*-Cys significantly reduced the POD and PPO activities, which agreed with the results in button mushroom, lotus root, litchi fruit and *Pholiota nameko* mushroom [8,9,10]. The anti-browning effects of *L*-Cys may result from the unique physicochemical characteristic and the triggering of the H_2_S biosynthesis [7].

This study demonstrated that exogenous *L*-Cys treatment increased the amino acid levels and the respective metabolism in fresh-cut Changgen mushrooms, which were corroborated by transcriptome analysis (Figure 4A–C and Figure 7). Additionally, the enhancement of endogenous *L*-Cys by exogenous *L*-Cys was also observed in postharvest plums and apples [13,14]. Notably, the accumulated endogenous *L*-Cys provides abundant precursors for H_2_S biosynthesis.

Additionally, *L*-Cys treatment significantly increased OrCBS and OrCSE activity and expression levels to increase endogenous H_2_S production, which was verified by qPCR and transcriptome analysis (Figure 4D–F and Table 1). Previous studies revealed that *L*-Cys treated fresh-cut button mushrooms promoted endogenous H_2_S production during storage by increasing the AbCBS enzyme and decreasing AbCSE [7]. These results indicated the different roles of CBS and CSE gene expressions in H_2_S biosynthesis between mushroom species. It can be inferred that the exogenous *L*-Cys boosted the levels of endogenous *L*-Cys and Met and improved the genes expression and activities of OrCBS and OrCSE in fresh-cut Changgen mushrooms, thereby facilitating H_2_S biosynthesis [13].

The activities of mitochondrial ATPases, particularly H^+^-ATPase and Ca^2+^-ATPase, are crucial for maintaining the cellular microstructure and mitochondrial integrity, reducing mitochondrial swelling and increasing the ATP content and EC [41]. This study discovered that exogenous *L*-Cys effectively maintained the ATP, ADP and AMP levels and the EC (Figure 5). The observed effects may be attributed to the induced H_2_S level, which regulated the CCO, H^+^-ATPase and Ca^2+^-ATPase activity and corresponding gene expression levels (Figure 5), as discovered in nectarine and broccoli [22,23].

Enhancing antioxidant capacity and maintaining a stable level of ROS are critical for preventing senescence and prolonging the shelf life of fresh produce [38]. DPPH is a stable free radical. The elevated DPPH scavenging activity, coupled with the reduction in H_2_O_2_ and O_2_^−^, indicated an overall enhancement in antioxidant capacity (Figure 3C–E). The fresh produce has evolved an efficient antioxidant system to scavenge ROS to avoid oxidative damage caused by accumulated ROS, including SOD, CAT, POD and APX enzymes [42]. Metalloenzyme SOD catalyzes the conversion of ·O_2_^−^ into H_2_O_2_, and CAT removes the resultant H_2_O_2_. Meanwhile, the APX and POD are the key enzymes responsible for H_2_O_2_ scavenging during oxidative stress [39]. In parallel, GR, functioning as a flavoprotein oxidoreductase, facilitates the reduction in oxidized glutathione to the reduced form [43]. The present study discovered that *L*-Cys treatment significantly enhanced the activities of SOD, GR and APX in Changgen mushrooms. Similar results were also discovered in fresh-cut apple and tuberous roots, which was contributing to the removal of ROS in tissues and maintain the normal physiological function of cells [13,42]. The fresh-cut Changgen mushrooms treated with *L*-Cys significantly reduced the POD activities. A similar trend was also discovered in litchi fruit, which could be attributed to the inhibited membrane de-compartmentalization by *L*-Cys [8]. Additionally, the present study discovered that the fresh-cut Changgen mushrooms treated with *L*-Cys significantly reduced the CAT activity level, which was contrary to the results in fresh-cut apple and litchi [8,13]. This phenomenon may be attributed to the fact that APX exhibits a higher affinity for H_2_O_2_ and primarily functions as a reducing agent in multiple cellular organelles, whereas CAT is predominantly localized in peroxisomes and displays a relatively weaker affinity for H_2_O_2_ [44]. Similarly to button mushrooms, the present study provided an evident proof that H_2_S production participates in maintaining antioxidant capacity and ROS in fresh-cut Changgen mushrooms by *L*-Cys treatment [7].

Leaf senescence and fruit overripening are also significant problems during storage. Recent findings in fruits and vegetables have demonstrated that *L*-Cys delays leaf senescence in Chinese flowering cabbage by reducing chlorophyll degradation, maintaining ROS homeostasis and enhancing endogenous H_2_S production [45]. The application of *L*-Cys effectively delayed tomato fruit ripening by suppressing the biosynthesis of carotenoids and lycopene, inhibiting chlorophyll degradation and delaying the respiration peak. Additionally, transcriptome analysis revealed that numerous genes related to ethylene biosynthesis were down-regulated [46]. It is evident that there are numerous undiscovered application scenarios for *L*-Cys, as well as potential uses in various fruits and vegetables and the mechanisms behind preservation functions. Further in-depth studies are necessary to explore these areas. The current study, along with previous studies, have effectively recognized changes in substance composition content, genes expression and enzymes activity. Despite these findings, the study has not demonstrated clear-cut causal links between *L*-Cys treatment and the phenotypic effects that have been documented. This undermines the assertions of mechanistic insights. Future research must prioritize the integration of additional molecular-level studies, such as the exploration of transcription factors, the analysis of interaction between proteins and the application of gene-editing techniques. This will enable a deeper exploration of the storage effect and freshness-preserving capabilities of *L*-Cys.

## 5. Conclusions

**Figure 8:**
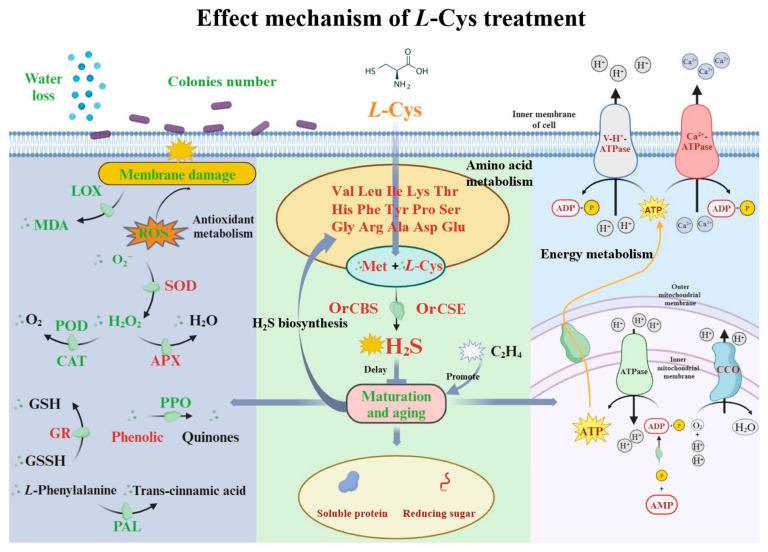
Mechanism map of *L*-Cys treatment on Changgen mushrooms. The red font represents an increase in the enzyme activity and the substance, the green font represents a decrease in the enzyme activity and the substance. Thin black arrows denote biological reactions, bold blue arrows signify promotion, and bold blue flat heads indicate inhibition. The purple capsule shape represents a colony.

In summary, fresh-cut Changgen mushrooms, a popular ready-to-eat product, face significant challenges in preservation due to their rapid quality deterioration. In the present study, the utilization of the natural preservative *L*-Cys has demonstrated promising potential in addressing these challenges. As shown in Figure 8, exogenous *L*-Cys treatment was effective in retarding the weight loss, browning degree, nutrition depletion and microbial contamination. Notably, exogenous *L*-Cys significantly stimulated the metabolism of 17 amino acids, among which the endogenous *L*-Cys and Met serveas the gaseous signaling molecule H_2_S biosynthesis precursors. Subsequently, it was discovered that an increase in endogenous H_2_S levels, as well as enzymatic activities and gene expressions of the OrCBS and OrCSE, was triggered. Furthermore, *L*-Cys enhanced energy metabolism by improving CCO, Ca^2+^-ATPase and H^+^-ATPase enzymes activity. Additionally, *L*-Cys treatment improved the ROS by regulating enzyme activities such as polyphenol oxidase, catalase and superoxide dismutase. This study contributed valuable insights to the mechanisms of the green preservation agent *L*-Cys on the fresh-cut edible mushroom.

## Figures and Tables

**Table 1:** Changes in the selected gene expression levels and the corresponding primers.

Gene Name	Gene ID	Gene Annotation by Swissprot	Regulations	log2foldchange		Primer Name	Sequence (5′–3′)
				qRT-PCR	RNA Seq		
*OrCBS*	MDBOrad1_07857	Cystathionine beta-synthase	Up	+1.73	+2.12	CBS F	GCAGGAAAACGACGATT
						CBS R	ACGACAGCACCACCACA
*OrCSE*	MDBOrad1_19093	Cystathionine gamma-lyase	Up	+6.61	+3.93	CSE F	CGAGAAGGACGGACAG
						CSE R	TTGCTACATCAACGGG
*OrCCO*	NewGene_3139	Cytochrome c oxidase subunit 1	Down	+7.46	+6.31	CCO F	GGAATATCCTTGGGGACT
						CCO R	AACGAGGTGAGGTTGTGA
*OrH^+^-ATPase 1*	MDBOrad1_01006	V-type proton ATPase subunit C	Down	−1.04	−1.10	H^+^-ATPase F 1	AACGACGATGGGCTA
						H^+^-ATPase R 1	CACGCATACGGGCTG
*OrH^+^-ATPase 2*	MDBOrad1_09920	V-type proton ATPase subunit D	Down	−1.85	−1.02	H^+^-ATPase F 2	TACTAAAGAAGGTTGAT
						H^+^-ATPase R 2	GTACGAGATAGGATATG
*OrH^+^-ATPase 3*	MDBOrad1_11764	V-type proton ATPase subunit a	Down	−1.32	−1.16	H^+^-ATPase F 3	GATCGGTTCGCATAGGT
						H^+^-ATPase R 3	TCTGGGGGATGAAGTTG
*OrCa^2+^-ATPase*	MDBOrad1_14841	Calcium-transporting ATPase 3	Up	+1.71	+2.20	Ca^2+^-ATPase F	CGAAGACAAGAATGGTG
						Ca^2+^-ATPase R	TGGTCAGGCAAATAGAG

## Data Availability

The original contributions presented in the study are included in the article, further inquiries can be directed to the corresponding author.

## Supplementary Materials

The following supporting information can be downloaded at: https://www.mdpi.com/article/10.3390/foods14030496/s1, Table S1: Changes in the selected gene expression levels by transcription sequencing.
