# Supplementary material for: Enhancing Postharvest Quality of Fresh-Cut Changgen Mushrooms by Exogenous L-Cysteine Treatment: Aspects of Accumulating Amino Acids, Triggering Energy Metabolism and Enhancing Endogenous H2S Regulation
Source: Foods. 2025 Feb 4;14(3):496. doi: 10.3390/foods14030496 (PMC11817185; doi:10.3390/foods14030496)
Supplement: Supplementary file 1 [file foods-14-00496-s001.zip › foods-3396328-supplementary.pdf]

Table S1. Changes of the selected gene expression levels by transcription sequencing.

Number	Gene ID	Gene Name	Regulation	Log2foldchange
1	MDBad1_12620	*LOX1*	Down	-3.35
2	MDBad1_06401	*LOX2*	Down	-1.49
3	MDBad1_20980	*PAL*	Down	-1.41
4	MDBad1_16795	*POD1*	Down	-1.56
5	MDBad1_11407	*POD2*	Down	-1.51
6	MDBad1_12619	*CAT1*	Up	+2.61
7	MDBad1_19481	*CAT2*	Up	+1.01
8	MDBad1_04981	*CAT3*	Up	+2.71
9	MDBad1_14132	*SOD1*	Up	+1.20
10	MDBad1_12760	*SOD2*	Up	+2.38
11	MDBad1_11683	*GR*	Up	+1.06
12	MDBad1_02449	*mannosyl-oligosaccharide 1, 2-alpha-mannosidase1*	Down	-1.27
13	MDBad1_08544	*mannosyl-oligosaccharide 1, 2-alpha-mannosidase2*	Down	-1.31
14	MDBad1_11531	*endo-1, 3-beta-glucosidase*	Down	-4.73
15	MDBad1_02726	*chitinase*	Down	-1.15
